# Influence of Seed Disinfection Treatments on the Germination Rate and Histamine-Degrading Activity of Legume Sprouts

**DOI:** 10.3390/foods13244105

**Published:** 2024-12-18

**Authors:** Judit Costa-Catala, Jaume Bori, M. Teresa Veciana-Nogués, M. Luz Latorre-Moratalla, M. Carmen Vidal-Carou, Oriol Comas-Basté

**Affiliations:** 1 Departament de Nutrició, Ciències de l’Alimentació i Gastronomia, Campus de l’Alimentació de Torribera, Universitat de Barcelona, Av. Prat de la Riba 171, 08921 Santa Coloma de Gramenet, Spain; jcostacatala@ub.edu (J.C.-C.); veciana@ub.edu (M.T.V.-N.); mariluzlatorre@ub.edu (M.L.L.-M.); mcvidal@ub.edu (M.C.V.-C.); 2 Institut de Recerca en Nutrició i Seguretat Alimentària (INSA·UB), Universitat de Barcelona, Av. Prat de la Riba 171, 08921 Santa Coloma de Gramenet, Spain; 3 Associated British Foods Ingredients (ABFI), Escoles Píes 49, 08017 Barcelona, Spain; jbori@dr-healthcare.com

**Keywords:** diamine oxidase (DAO) enzyme, histamine, histamine intolerance, legume sprouts, seed disinfection, germination rate, catalase enzyme

## Abstract

Edible legume sprouts have been proposed as a promising plant-based source of the enzyme diamine oxidase (DAO), which plays a key role in degrading histamine at an intestinal level and preventing the development of histamine intolerance symptoms. However, the temperature and humidity conditions required for seed germination can also favor the rapid growth of yeast and mold, potentially compromising sprout yield and quality. The aim of this study was to evaluate the influence of different seed disinfection treatments on both the germination rate and DAO enzymatic activity in sprouts of four *Leguminosae* species. Seed disinfection with 70% ethanol for either 5 or 15 min slightly increased the germination rates of chickpea and soybean sprouts without affecting DAO activity, regardless of treatment duration. However, in lentil and green pea sprouts, ethanol disinfection caused a statistically significant reduction in histamine-degrading capacity. In contrast, treating seeds with sodium hypochlorite for 15 min increased germination rates by up to 14% and preserved DAO activity in all legume sprouts tested. These results indicate that incorporating a seed disinfection step during legume sprouting may affect both the DAO enzymatic activity and germination rate.

## 1. Introduction

Diamine oxidase (DAO, EC 1.4.3.22), also referred to as histaminase, is a copper-containing enzyme that catalyzes the oxidative deamination of the primary amino group of histamine (2-(1*H*-imidazol-4-yl)ethanamine) and other diamines, converting them into their corresponding aldehydes and generating stoichiometric amounts of ammonia and hydrogen peroxide [1]. DAO is widely distributed across microorganisms, plants, and mammals [2] and it plays a crucial role in the degradation of diamines from food in human intestines [3].

Histamine intolerance, a food-related disorder, occurs when the degradation of histamine in the intestine is impaired by reduced DAO activity, resulting in an increase in its accumulation in plasma, and the subsequent onset of adverse health effects [4]. Clinical manifestations of histamine intolerance include a wide range of non-specific gastrointestinal and extraintestinal symptoms and usually appear in susceptible individuals after the consumption of foods containing moderate or even small amounts of histamine and/or other biogenic amines [5,6].

In addition to following a low-histamine diet, current strategies to prevent histamine-related symptoms include enhancing intestinal histamine degradation through dietary supplementation with gastrointestinal tablets containing exogenous DAO [7]. These commercial DAO supplements are mainly formulated using porcine kidney protein extract, a DAO-containing active ingredient approved both as a food supplement and as food for special medical purposes by the European Commission in 2017 [8,9].

Recently, plant-based DAO has emerged as a promising alternative to animal DAO for treating histamine-related disorders [10,11]. Sprouted seeds from certain *Leguminosae* species have been identified as significant sources of DAO, showing similar or even higher in vitro histamine-degrading activity compared to animal-derived DAO [10,12,13]. Seed germination notably enhances DAO activity, likely due to the role of the enzyme in modulating the cell wall architecture during plant development and in defense against pathogens [14,15].

Reports in the literature indicate that various germination conditions, such as temperature, duration, and light exposure, can significantly influence the DAO activity of legume sprouts [10,12,15]. For example, Comas-Basté et al. [10] demonstrated that etiolated legume sprouts (germinated in darkness) exhibited higher histamine catalytic activity compared to those grown under light conditions.

However, the temperature and humidity required for optimal seed germination can also create favorable conditions for the rapid growth of yeast and mold, potentially compromising sprout yield and quality. To address this, pre-germination seed disinfection treatments are commonly used in the sprouting agrifood industry. However, there are currently no available data on the impact of these sanitation treatments on the DAO enzymatic activity of the sprouts.

As mentioned, the histamine-degrading activity of DAO generates hydrogen peroxide as a by-product, which in excess can lead to oxidative damage to cell structures [1,16,17]. Catalase breaks down hydrogen peroxide into water and oxygen, potentially reducing oxidative stress. In this context, some researchers have proposed that the concomitant presence of catalase and DAO activity in plant-based active ingredients may help to mitigate the progressive accumulation of hydrogen peroxide at the intestinal level [1].

The primary aim of this study was to evaluate the effects of different disinfection treatments on both DAO enzymatic activity and germination rates in sprouts of four *Leguminosae* species. Additionally, the catalase activity of the lyophilized legume sprouts was assessed.

## 2. Materials and Methods

### 2.1. Chemicals

Hydrochloride acid 0.1 M, brij^®^ L23 solution and phthaldialdehyde (OPA) were acquired from Merck (Darmstadt, Germany). Sodium di-hydrogen phosphate anhydrous, di-sodium hydrogen phosphate anhydrous, sodium hypochlorite, ethanol, hydrogen peroxide 30%, sodium acetate anhydrous and perchloric acid were obtained from PanReac Química (Castellar del Vallès, Spain). Potassium hydroxide, 2-mercaptoethanol, 1-octanesulfonic acid sodium salt, acetic acid, boric acid, histamine dihydrochloride, methanol and acetonitrile were purchased from Sigma-Aldrich (St. Louis, MO, USA). Ultrapure water (18.2 MΩcm) was generated using a LaboStar System from Evoqua Water Technologies (Warrendale, PA, USA).

### 2.2. Legume Species

Four edible species of the *Leguminosae* plant family were included: chickpea (*Cicer arietinum* L.), lentil (*Lens culinaris* Medik.), soybean (*Glycine max* (L.) Merr.) and green pea (*Pisum sativum* L.). These legume seeds were acquired from local suppliers and stored in a cool and dry refrigerated chamber at 5 ± 2 °C and a relative humidity of 55 ± 2% (Coref, Montgat, Barcelona), avoiding sudden temperature changes. The seeds were kept inside their original opaque container, which was properly sealed to protect them from moisture and light. The maximum storage time between purchasing the seeds and their germination was one month.

### 2.3. Seed Disinfection Treatment

A total of 250 g of seeds from each legume species was disinfected in 500 mL of an aqueous solution containing (a) 70% ethanol or (b) sodium hypochlorite at two different concentrations (70 and 100 mg/L). A control batch was treated with distilled water following the same procedure. Each disinfection treatment was applied for two durations (5 and 15 min). The seeds were placed in a beaker and agitated continuously in the disinfectant solution, and then were strained and rinsed five times with distilled water prior to germination.

### 2.4. Germination of Seeds

After undergoing the different disinfection treatments (Section 2.2), the seeds were soaked overnight in distilled water at room temperature in darkness. After soaking, the seeds were rinsed, strained, and placed in an inert cotton substrate for germination in a climate-controlled chamber (Memmert^®^, Memmert GmbH + Co. KG, Schwabach, Germany). The germination conditions were as follows: 5 days at 30 °C, 70% relative humidity, and darkness [10]. During the germination process, the seeds were sprayed with distilled water twice a day. After five days, the sprouts were harvested and then frozen at −80 °C in an ultra-low temperature freezer (NU-99728J, NuAire, Plymouth, MN, USA). Next, the sprouts were freeze-dried at a chamber pressure of 0.22 mbar, with a temperature increase from −85 °C to 22 °C over 48 h (Cryodos-50, Telstar, Terrassa, Spain). The freeze-dried sprouts were then ground in a mortar to obtain a homogeneous product.

To determine the germination rate for each experimental condition, a ratio (in percentage) was calculated between the weight of fresh sprouts obtained after germination and the weight of dry seeds used as raw material.

### 2.5. Determination of DAO Activity

The in vitro enzymatic DAO activity was measured following the protocol outlined by Comas-Basté et al. [18]. This procedure assesses histamine degradation during the oxidative deamination process mediated by the DAO enzyme, employing an enzymatic assay coupled with ultra-high-performance liquid chromatography and fluorescence detection (UHPLC-FL).

For the assay, 10 mg of lyophilized legume sprout was mixed with 20 mL of 50 mM phosphate-buffer solution (pH 7.2) and incubated in a shaker (NB-T205, N-BIOTEK, Inc., Bucheon-si, Republic of Korea) at 37 °C and 200 rpm for 30 min. The reaction was initiated by adding 45 μM of a histamine standard solution, and the mixture was continuously incubated (37 °C, 200 rpm). Aliquots of 500 μL were extracted each 60 min for the first four hours to monitor the degradation of histamine throughout the reaction. At each point of analysis, 15 μL of 2 N perchloric acid solution was added to cease the enzymatic reaction, and the sample was homogenized and centrifuged for 5 min (15,000 rpm, 4 °C). The supernatant was passed through a 0.22 µm GHP filter and kept at 4 °C until UHPLC-FL analysis.

#### UHPLC-FL Analysis

Histamine determination was performed using ion-pair reverse-phase UHPLC-FL, incorporating online post-column derivatization with OPA following the method described by Latorre-Moratalla et al. [19]. UHPLC-FL was carried out using a Waters Acquity^TM^ Ultra Performance Liquid Chromatography apparatus equipped with a quaternary pump, an auto-sampler and a fluorescence detector. For the post-column derivatization of histamine, an additional pump was connected to a zero-dead-volume mixing T positioned between the column outlet and the fluorescence detector. Chromatographic separation was achieved on an Acquity UPLC BEH C18 column (1.7 µm, 2.1 mm × 50 mm) (Waters Corp., Milford, MA, USA), which was kept in an oven to maintain a consistent temperature (42 °C). Data acquisition and processing were performed with Empower^TM^ 3 software (Waters Corp., Milford, MA, USA).

The chromatographic conditions were as follows: the mobile phase was delivered at a flow rate of 0.8 mL/min, while the derivatization reagent was pumped at 0.4 mL/min. The specific composition of the mobile phase and of the OPA derivatization reagent was found by Latorre-Moratalla et al. [19]. An automatic injection of 1 µL of both the standard solution and samples was performed. Fluorimetric detection was conducted with an excitation wavelength of 340 nm and an emission wavelength of 445 nm. An example of an UHPLC-FL chromatogram is provided in the Supplementary Materials (Supplementary Figure S1).

The DAO enzymatic activity corresponds to the slope of the line obtained by plotting the evolution of the remaining histamine amount (in nmol) along the different sampling times (in minutes). This DAO activity is divided by the amount of the lyophilized legume sprout sample to obtain the specific enzymatic activity, which is expressed in mU/mg, corresponding to the amount of histamine degraded by one milligram of lyophilized legume sprout per minute (nmol of degraded histamine per minute/mg of sample).

### 2.6. Determination of Catalase Activity

The analysis of catalase activity was performed by monitoring the rate of disappearance of a known amount of hydrogen peroxide following the methodology developed by Leonida et al. [17]. Briefly, 10 mg of the sample was homogenized with 500 μL of 0.05 M phosphate buffer (pH 7.2) using a vortex shaker for 30 min at room temperature. The samples were then centrifuged for 20 min at 14,000 rpm and 20 °C. The catalase activity assay was performed by mixing 10 μL of this sample mixture with 2.99 mL of a catalase reaction solution consisting of 15 mM hydrogen peroxide (30%) in 50 mM phosphate potassium buffer (pH 7.2). The disappearance of hydrogen peroxide was monitored by measuring absorbance at 240 nm for 10 min, using 0.05 M phosphate buffer as a blank. Catalase activity was expressed in nmol/min/mg.

### 2.7. Statistical Analysis

Data analysis was carried out using IBM SPSS Statistics 27.0 software (IBM Corporation, Armonk, NY, USA). The results are shown as mean values ± standard deviation (mean ± SD) from two independent experiments performed in duplicate. To evaluate the statistical significance of changes in enzymatic activity across different conditions, the nonparametric Mann–Whitney U test was used. Differences were deemed statistically significant at *p* < 0.05.

## 3. Results and Discussion

### 3.1. Influence of Different Seed Disinfection Treatments

**Figure 1:**
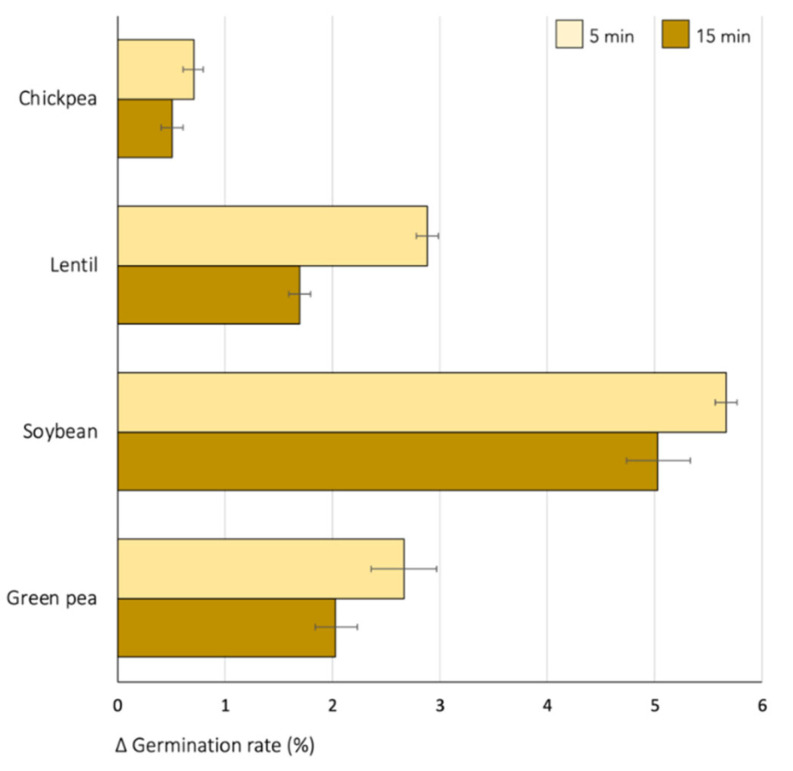
Effect of seed disinfection with 70% ethanol on the germination rate of four species of lyophilized legume sprouts. The horizontal axis shows the change in germination rates following seed disinfection compared to sprouts obtained from untreated seeds.

Disinfection of seeds with 70% ethanol resulted in a slight increase in germination rates. Figure 1 shows the changes in seed germination after applying this treatment for either 5 or 15 min compared to untreated seeds. The effect on the germination rate varied depending on the cultivated species, with the most significant improvement observed in ethanol-treated soybean seeds, which showed a 6% increase in fresh sprout weight. In contrast, lentil and green pea sprouts showed only a 2–3% increase in the germination rate compared with the non-treated seeds, which was even lower in the case of chickpeas (approximately 0.5%).

The duration of the sanitizing treatment is another important variable. In all cases, the longer application time was associated with a smaller increase in germination, which is a pattern observed in other studies. For example, S. Santos et al. [20] obtained satisfactory germination rates in different lentil varieties when using 70% ethanol for 5 min. In contrast, Afzal et al. [21] reported potential toxic effects of prolonged ethanol exposure in tomato seeds, where sprout length decreased after 24 h of treatment, even at lower concentrations.

**Figure 2:**
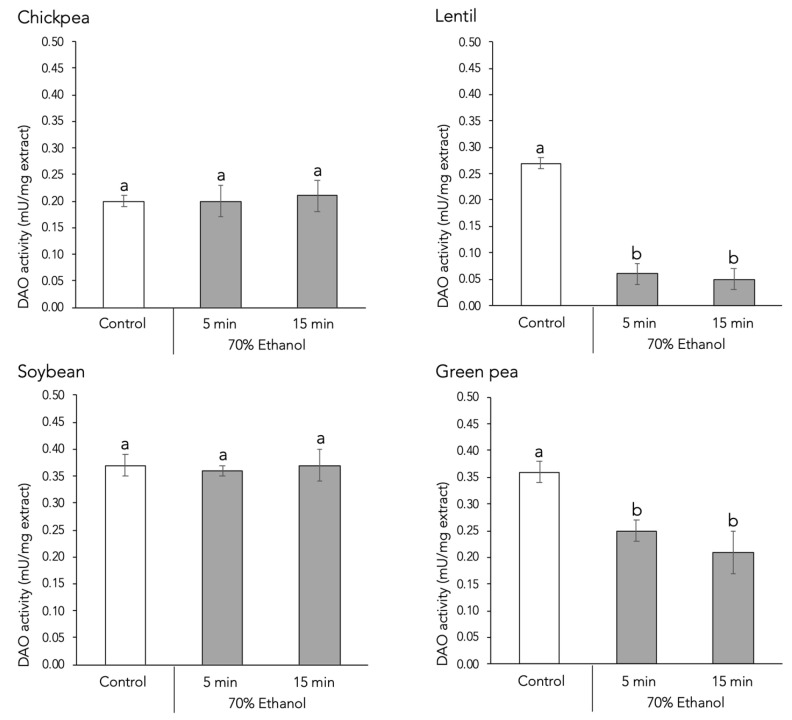
Effect of 70% ethanol seed disinfection treatment on the DAO enzymatic activity in four species of lyophilized legume sprouts. Different letters denote statistically significant differences (*p* < 0.05) between control and disinfection treatments.

Regarding in vitro histamine-degrading activity, ethanol treatment had no observable effect, either positive or negative, on chickpea and soybean sprouts regardless of the application time (Figure 2). However, ethanol seed disinfection led to a statistically significant reduction (*p* < 0.05) in DAO activity in lentil and green pea sprouts when applied for 5 or 15 min, with no statistically significant differences between the two durations. The negative effect of ethanol was especially pronounced in lentil sprouts, where DAO activity decreased by approximately 80% compared to the control batch. The differing responses of plant species to ethanol disinfection are likely due to variations in seed size and surface contact area, with smaller seeds like lentils being most affected due to their higher surface-area-to-volume ratio. To date, little research has explored the effect of ethanol seed disinfection on enzymatic activities in sprouts, and no research has focused on DAO. In 2013, Afzal et al. [21] reported that applying 6% ethanol for 24 h reduced both catalase and peroxide dismutase activities in tomato seeds.

**Figure 3:**
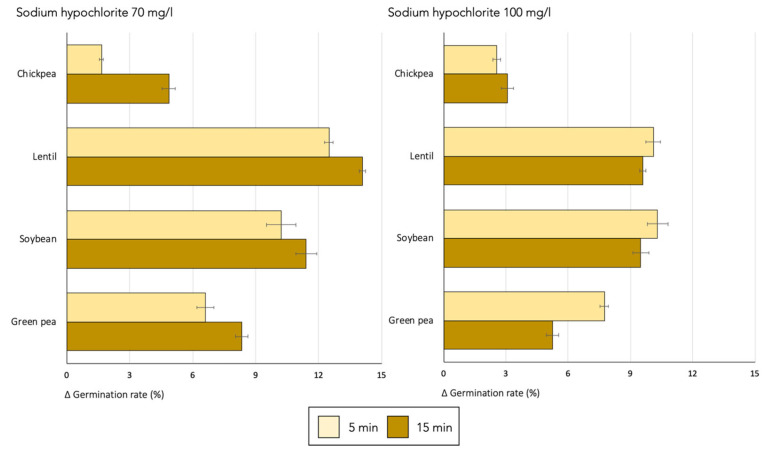
Effect of seed disinfection with sodium hypochlorite at two concentrations, 70 mg/L and 100 mg/L, on the germination rate of four species of lyophilized legume sprouts. The horizontal axis shows the change in germination rates following disinfection compared to untreated seeds.

The prior disinfection of seeds with sodium hypochlorite increased germination rates in all the legume species studied (Figure 3). Similarly to the ethanol treatment, the effects varied depending on the seed species and disinfection time; however, in this case, the concentration of the disinfectant was also an influential factor. In all species, sodium hypochlorite had a more pronounced effect on germination rates than 70% ethanol. As in the ethanol treatment, the smallest increases in the germination rate were observed in chickpeas and green peas with gains of up to 5% and 8%, respectively, compared to 11% and 14% in soybean and lentil seeds, respectively.

When comparing the two concentrations of sodium hypochlorite, 70 mg/L generally resulted in a better germination performance compared to the control. At this concentration, the longer treatment time led to a greater increase in germination rates, especially in chickpeas and green peas (*p* < 0.05). At the higher concentration of 100 mg/L, extending the treatment time did not significantly affect the germination rate except in green peas, where a marked reduction was observed (up to 32%). As is in the case of ethanol, there are few studies in the scientific literature that have investigated the influence of sodium hypochlorite on the germination rate of legume sprouts. Tornuk et al. [22] reported an improvement in the germination rate of wheat seeds (*Triticum aestivum*) after 30 min of disinfection with sodium hypochlorite at 100 mg/L and 200 mg/L. However, this positive effect was lost when the concentration was increased to 400 mg/L.

**Figure 4:**
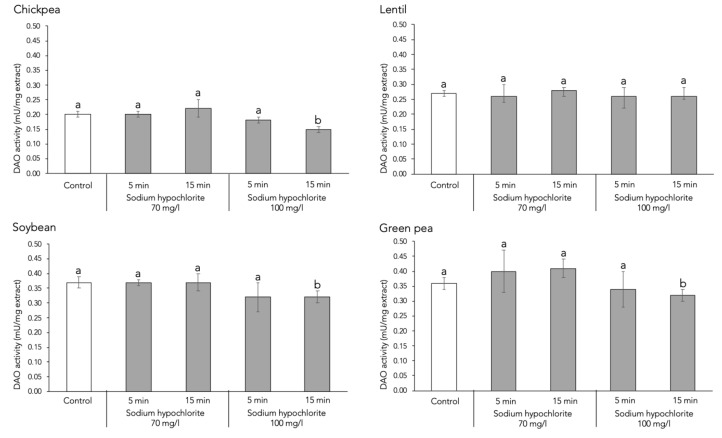
Effect of sodium hypochlorite seed disinfection on DAO enzymatic activity in four species of lyophilized legume sprouts. Different letters denote statistically significant differences (*p* < 0.05) between control and disinfection treatments.

Regarding DAO activity, treating seeds with sodium hypochlorite at 70 mg/L for 5 or 15 min had no effect on the in vitro histamine-degrading capacity of any legume sprouts studied (Figure 4). Similarly, applying 100 mg/L of sodium hypochlorite for 5 min did not influence DAO activity. However, when the disinfection time was extended to 15 min at this higher concentration, DAO activity not only failed to increase, but even decreased in three of the four legume species (*p* < 0.05). It is possible that sodium hypochlorite, at certain concentrations and exposure times, alters the properties of cell membranes, which may adversely affect cellular metabolic activities [23]. However, little is known about the biochemical impact of sodium hypochlorite on enzymatic activity. A different scenario was observed by Kaneko and Morohashi, who reported an induction of α-amylase activity in the cotyledons of mung beans (*Vigna radiata* (L.) Wilczek) and green peas after an 8 min seed disinfection with sodium hypochlorite at concentrations up to 25-fold higher than those in the present study [23]. Notably, these concentrations far exceed the maximum levels authorized for sodium hypochlorite use as a disinfectant in vegetal foods [24].

In the production of edible sprouts, selecting an effective disinfection agent, along with the appropriate treatment parameters (time and concentration), is essential for ensuring optimal plant growth and high germination rates and controlling the microbial load. No single disinfection method is universally suitable for all plants [25]. Some studies have employed aqueous solutions of 70% ethanol and various concentrations of sodium hypochlorite as surface disinfectants for seed germination [21,22,26]. Both ethanol and sodium hypochlorite not only inhibit mold growth during germination but are also reported to stimulate germination or break seed dormancy. For example, Kaneko and Morohashi [23] found that sodium hypochlorite can partially erode the seed coat, thereby enhancing its permeability to oxygen. The results obtained in the current study corroborate the potential of ethanol and sodium hypochlorite to stimulate the germination of the four tested legume seeds.

Overall, the disinfection process can be expected to have significant effects on germination performance and even seed metabolism. Therefore, selecting the most suitable seed hygienic treatment is essential to maximize germination rates while preserving the enzymatic functionality of the resulting sprouts. According to the results from this study, seed disinfection with 70 mg/L of sodium hypochlorite for 15 min enhanced the germination rate of legume sprouts without negatively affecting DAO enzymatic activity.

### 3.2. Catalase Activity

In this study, catalase activity was measured in the lyophilized sprouts of four legume species obtained after germination, following prior disinfection with 70 mg/L of sodium hypochlorite for 15 min. This treatment was chosen as it resulted in a higher germination rate without adversely affecting the DAO activity of the sprouts.

All tested lyophilized legume sprouts exhibited catalase activity, with variations observed among the four species. Green pea sprouts showed the highest catalase activity, with values up to 4-fold higher than those of the other species. This finding is consistent with previous research by Luhová et al. [27], who observed similar levels of catalase activity in sprouts from 13 different green pea cultivars germinated in darkness. Furthermore, Luhová et al. noted that this enzymatic activity increased 1–3-fold when the seeds were germinated under a 12 h photoperiod [27]. This pattern contrasts with the behavior of the DAO enzyme, which has been shown to become significantly more active in sprouts germinated in darkness [10]. Regarding the impact of seed disinfection, treatment with an aqueous solution of 70 mg/L sodium hypochlorite for 15 min did not affect the catalase activity of the lyophilized sprouts (Table 1) (*p* > 0.05).

## 4. Conclusions

Overall, these results demonstrate that incorporating a seed disinfection treatment step during the germination process of edible legume sprouts can significantly influence both the germination rate and the activity of the DAO enzyme. Specifically, while prior seed disinfection with 70% ethanol for either 5 or 15 min negatively impacted the histamine-degrading activity of certain sprouts, treatment with an aqueous solution of 70 mg/L sodium hypochlorite for 15 min increased the germination yield without impairing DAO activity. Additionally, this sanitizing method preserved catalase activity in all tested lyophilized legume sprouts, with notably high activity observed in green pea sprouts. These novel findings regarding the effects of seed disinfection on DAO activity in edible legumes are particularly valuable for the production of plant-based DAO. This enzyme is a promising active ingredient for formulating food supplements aimed at the dietary management of histamine intolerance. Furthermore, the preservation of catalase activity suggests potential additional benefits, as catalase is known for its role in protecting cells from oxidative damage. Therefore, the implementation of an effective seed disinfection protocol using sodium hypochlorite (70 mg/L) not only enhances germination rates but also maintains the histamine-degrading activity of legume sprouts.

## Figures and Tables

**Table 1:** Catalase activity (mean ± standard deviation) of lyophilized legume sprouts obtained with and without (control) a prior seed disinfection treatment using sodium hypochlorite at 70 mg/L for 15 min.

	Catalase Activity (nmol/min/mg)		
	Control	70 mg/L Sodium Hypochlorite for 15 min	*p*-Value
Chickpea	15.14 ± 6.30	15.37 ± 2.61	*p* = 0.928
Lentil	37.84 ± 0.97	35.09 ± 3.83	*p* = 0.106
Soybean	30.98 ± 2.43	31.19 ± 4.88	*p* = 0.805
Green pea	65.02 ± 2.10	56.65 ± 11.43	*p* = 0.153

## Data Availability

The original contributions presented in the study are included in the article/Supplementary Materials; further inquiries can be directed to the corresponding author.

## Supplementary Materials

The following supporting information can be downloaded at: https://www.mdpi.com/article/10.3390/foods13244105/s1, Figure S1: Overlapping chromatograms of histamine (initial concentration of 45 μM) at the starting point (black) and after 1 h (dark blue), 2 h (green), 3 h (light blue) and 4 h (pink) of the reaction for a sample of lyophilized green pea sprouts.
