# Supplementary material for: Influence of Seed Disinfection Treatments on the Germination Rate and Histamine-Degrading Activity of Legume Sprouts
Source: Foods. 2024 Dec 18;13(24):4105. doi: 10.3390/foods13244105 (PMC11675340; doi:10.3390/foods13244105)
Supplement: Supplementary file 1 [file foods-13-04105-s001.zip › foods-3343724-Supplementary_Figure S1.pdf]

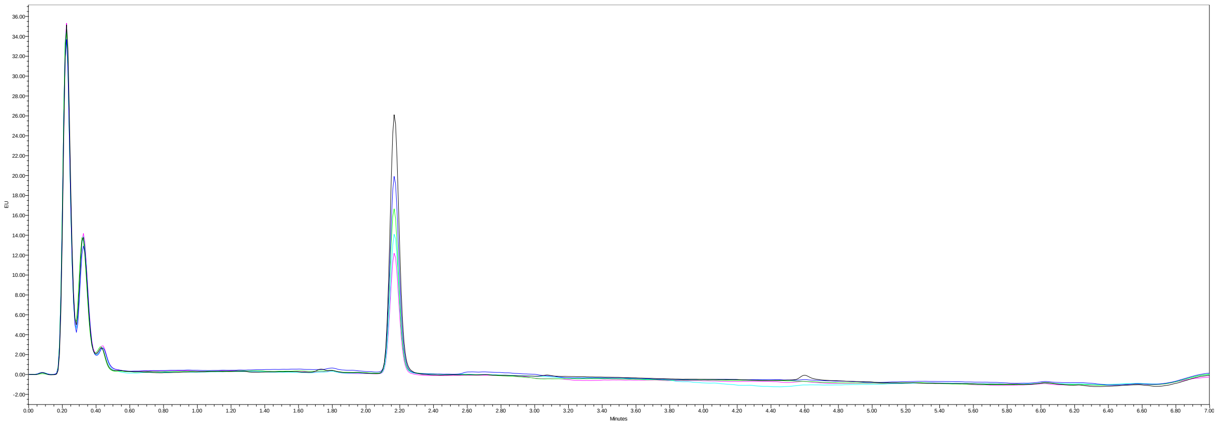

Figure S1. Overlapping chromatograms of histamine (initial concentration of 45 μM) at the starting point (black) and after 1 h (dark blue), 2 h (green), 3 h (light blue) and 4 h (pink) of reaction for a sample of lyophilised green pea sprouts.
